# Determinants of Mental Healthcare-Seeking Behavior of Postpartum Women in Ibadan, Nigeria

**DOI:** 10.3389/fgwh.2022.787263

**Published:** 2022-06-29

**Authors:** Oyeteju T. Odufuwa, Olanrewaju Olaniyan, Sam A. Okuonzi

**Affiliations:** ^1^Department of Economics, University of Ibadan, Ibadan, Nigeria; ^2^Health Policy Training and Research Programme, Department of Economics, University of Ibadan, Ibadan, Nigeria; ^3^African Centre for Global Health and Social Transformation (ACHEST), Kampala, Uganda

**Keywords:** Edinburgh postnatal depression scale, maternal mental health, women, healthcare-seeking behaviors, postpartum depression (PPD)

## Abstract

The mental healthcare-seeking behavior of postpartum mothers has received little attention in Nigeria. Previous studies in the country have focused on determinants of physical health-seeking behavior, choice of maternal healthcare provider, prevalence, and determinants of maternal mental illness, yet, determinants of maternal mental health-seeking behavior among Nigerian women has been understudied. This study, therefore, examined the determinants of mental health-seeking behavior among postpartum women in Ibadan, Nigeria. Maternal mental illness, which was proxied using postpartum depression, was computed using the Edinburgh Postpartum Depression Scale. Data for the study were obtained through a survey method using a 9-page questionnaire. A 3-stage sampling technique was employed. The first stage was a stratified sampling to disaggregate the health facilities offering postnatal and immunization services on the basis of ownership of public and private healthcare providers. In the second stage, seven healthcare facilities comprising three (3) private and four (4) public healthcare were purposively selected based on the number of attendees. The final stage was a random selection of 390 postpartum mothers attending postnatal and immunization clinics across seven healthcare facilities. The prevalence of depression among the mothers was 20.8%. While only 39.5% of the depressed women sought care, 22.3% of the non-sufferers also sought mental healthcare. This revealed that both sufferers and non-sufferers sought mental healthcare. Also, a higher incidence of postpartum depression among the sufferers increased the likelihood of seeking mental healthcare. Age, family history of postpartum depression, and having the desired gender of child were determinants of mental health-seeking behavior. Among the sufferers of postpartum depression who failed to seek care, a low perceived need for mental healthcare, the perception that the depressive symptoms will go on their own, as well as fear of being stigmatized as a “weak mother”, were reasons for not seeking mental healthcare. Thus, to promote mental healthcare, the non-cost factors, like availability and accessibility to a mental healthcare facility should be addressed. To achieve this, mental healthcare sensitization programs should be integrated into maternal healthcare at all levels, and mothers attending antenatal clinics should be routinely screened for early symptoms of depression in the postpartum period.

## Introduction

Health is integral to the attainment of economic growth and sustainable development of any nation (1). The total health and well-being of an individual are ultimately dependent not only on the physical but also on the mental health status of its people (2). However, more attention has been paid on the physical health of the populace (1), while the mental aspect has often been neglected, especially in developing countries (Nigeria inclusive).

In Nigeria, an estimated 20 to 30% of its over 200 million population is believed to suffer from mental illness (3). Studies on mental health in the country had focused on the prevalence of mental illnesses (4–6), its risk factors (7, 8), and the perception and attitude of people to mental illnesses, especially stigmatization (9). The aspect of mental health-seeking behavior has been grossly understudied both in Nigeria and other parts of the world (10).

Specifically for women, the most common mental illnesses experienced in the postpartum period are postpartum blues, depression, anxiety, and psychosis (11, 12). Approximately 10 to 20% of pregnant and postpartum women experience any of these mental health problems; primarily depression and /or anxiety during pregnancy or after childbirth (13–15). This rate is often higher in developing countries where about 15.6 and 19.8% of pregnant and new mothers, respectively, experience psychiatric disorders. Norhayati (16) observed a postpartum period depression (PPD) prevalence ranging between 1.9 and 82.1% globally; 2.2 and 74% in developed and developing countries, respectively.

Postpartum period depression, which is one of the most common maternal mental illnesses, has been identified as a worldwide public health issue and the leading contributor to the disease burden in women of reproductive age (17). In Nigeria, a PPD prevalence rate of 14.6% has been observed in the South West (5); 22.9 and 34.6% in Enugu, South East (6, 8), and 21.8% in Jos, North Central (18). This high prevalence rate of PPD in the country, the accompanying consequences of this invisible illness on the mother and infant, coupled with the few neuropsychiatric healthcare facilities (eight) in the country, is a cause for concern (5, 6).

Globally, the provision of mental healthcare services to the citizenry has been identified as one of the responsibilities of government (19). Yet, the Nigerian government lags in this role despite its whopping number of cases. The current mental health system in Nigeria is characterized by grossly inadequate mental health facilities (only eight federal neuropsychiatric hospitals exist in the nation), human resource inadequacy, and failure to implement the existing Mental Health Policy (20).

There is a dearth of studies on mental health-seeking behavior in Nigeria. Most studies reveal that women experiencing mood disorders in the postpartum period rarely seek help (10, 12, 21–24). In addition, in most countries, Nigeria inclusive, women hardly attend the scheduled six (6) weeks postnatal check-up (25).

The peculiarity of mental healthcare can be seen in 3-folds: the first is the need for long-term consistent treatment of the affected women (26). The second is the human resource need for treating mental disorders which also require a different cohort of professionals to provide adequate care for the patient (20). The third is the stigma associated with people undergoing mental care (24, 27).

Age, marital status, education, household headship, size, and expenditure were identified as determinants of health-seeking behavior among Nigerians (1). It was also observed that members of the household preferred first to consult traditional, patent medicine vendors (PMVs), and religious bodies (churches/mosques) before consulting healthcare professionals (HCPs). In addition, Murithi (28) has identified gender, quality of service, information about this quality, user fees, and wealth as determinants of the demand for healthcare in a rural slum in Nairobi, Kenya. Olasehinde and Olaniyan (29) have also found that prime-age adults had lower chances to seek care than children and old-age. Hence, adults in their prime years, regardless of gender, are less likely to seek care.

Household size, distance, the total cost of healthcare, and the quality of access routes were found as determinants of healthcare services in Kogi State, Nigeria (30). Household headship and level of education were found to be vital determinants of seeking healthcare during illness in Kwara State, Nigeria (31). Gender was also observed to play an important role in the decision of individuals to seek care in Edo State, Nigeria, as older men were seen to have poor health literacy and health-seeking pattern (32).

Proximity to a facility, quality of care received, insurance coverage, income, health status, education, age, and gender were found to be predictive factors in seeking healthcare in rural China (33). These factors were also affirmed by Gotsadze, et al. (34) in Georgia who further revealed that personal status, means of payment, level of education, and religious beliefs were factors that could deter or encourage an individual to seek healthcare during illness.

Maternal mental health problems are often undiagnosed, untreated, or undertreated (15). Often, mothers, despite having access to healthcare practitioners, fail to seek healthcare, and even if they do, they do not receive the appropriate healthcare (24, 35). James and Blank (34) and Smith (36) also found stigmatizing attitudes toward mental health, unfamiliarity with the signs and symptoms of perinatal mental illness, and insufficient knowledge among healthcare providers, which all contributed to the delay to seek mental healthcare. Rural residence and being divorced or unmarried (marital status) were found to be associated with lower use of mental health services in a community in Northwestern China (37). The failure of people to seek healthcare in cases of mental illness over a lifetime increased the prevalence of mental disorders by 21%, while the lifetime use of mental health services reduced the prevalence to 2.45 and 4.67%.

Factors affecting mental health-seeking behavior in the United Kingdom include expectations and experiences of mental healthcare professionals, the cultural needs, uncertainty of the role of professionals in providing the needed support, the perception that available healthcare was ineffective and inappropriate, hesitancy to approach professionals they were not familiar with, lack of continuity of care, and knowledge and experiences of healthcare during pregnancy (24). Bruffaerts, et al., (38) affirmed the case of “the worried well” who sought mental health treatment when they did not meet the criteria for a mental disorder. Hence, for PPD among mothers, most of the affected women do not seek care, while some who seek care are not diagnosed with mental illness.

In Nigeria, physical access to general and specialized mental healthcare, which is provided by the government, is limited to eight locations of the clinics, the few departments of psychiatry in the teaching hospitals and the privately-owned neuropsychiatric clinics, making it inequitable. This is quite worrisome. The limited access to mental health services impacts negatively on the decision of patients and their families to seek or not to seek mental healthcare.

This study was therefore aimed at examining the mental health-seeking behavior of postpartum women and the determinant factors of the decision to seek or not seek care. The hypothesis is that given a relatively high prevalence of PPD (as a proxy for mental illness during postpartum in Nigeria), the demand for mental healthcare is proportionally much less than it is expected to be. The objectives of this study are to (1) determine the factors making the sufferers of PPD seek or not seek mental healthcare, (2) determine the proportion of PPD sufferers and non-sufferers who seek mental healthcare, and (3) identify cost- and non-cost related factors preventing women suffering from PPD from seeking mental healthcare.

## Methods And Study Design

### Sample and Procedure

#### Target Population

A facility-based survey was conducted among mothers with babies below 18 months, attending postnatal and immunization clinics in Ibadan, the capital of Oyo State, Nigeria. Ibadan, which is one of the highly populated metropolitan cities in Nigeria, has one of the highest postnatal attendances in the country (25). The choice of mothers with children below 18 months is due to the proposition that there are cases of PPD that present at birth and extend beyond the first year of birth (8, 23). Participants were contacted at the clinics and were invited to participate in the study by filling out the questionnaires with the aid of the research team.

#### Sampling Frame

A three-stage sampling technique was employed. The first stage was a stratified sampling to disaggregate the health facilities offering postnatal and immunization services, on the basis of ownership, into public and private healthcare providers. Free immunization services are provided in public healthcare facilities in the country, while private facilities provide paid immunization services that extend from birth to beyond 18 months. In the second stage, seven healthcare facilities comprising three (3) private and four (4) public healthcare facilities were purposively selected based on clinics with the highest number of attendees (patronage). The final stage was a random selection of 390 women whose children were between 2 weeks and 18 months, across the seven (6) healthcare facilities based on their willingness to participate in the study.

#### Ethical Considerations

Ethical approval for this study was obtained from the Research and Ethical Committee of the University of Ibadan/University College Hospital Ethical Review Board (UI/UCH ERB) with the number NHREC/05/01/2008a in Ibadan, Oyo State, Nigeria. Consent was also obtained from the management of each hospital used in the study. The participation of the respondents was voluntary and based on verbal and written informed consent. They were also informed about their right to withdraw from participation if the need arose. The collected information was kept confidential and all data were anonymized, which minimized the risk of integrity infringement.

#### Data Collection Procedure

Data for the study were collected from mothers who brought their children for immunization services in the seven (6) healthcare facilities used. In this study, for the private healthcare providers, most of the immunizations were paid for, including the special vaccines. However, for the public clinics, all the vaccines were provided free of payment. As such, most rural mothers would rather visit the public healthcare facilities, while the mothers who were covered by the community-based or personal health insurance utilized the private facilities.

The administration of the research instruments was done in three phases. The first phase, which took place in June 2019, was to conduct a pilot study using the research instruments. The second phase was conducted for about 4 weeks in August 2019 after which a 12-week break was taken to ensure the availability of new cohorts of mothers at the immunization clinics. This was necessary because most immunizations were given at 4 weeks intervals. The final phase was conducted between December 2019 and January 2020. A five-man research team (comprising two student nurses, 2 field officers, and the Principal Investigator) who could speak Yoruba, the indigenous language fluently, was recruited to assist in the questionnaire administration. The response rate was about 75% across the seven (7) clinics. The clinical information of the mothers was not explored in this study because for most of the sampled mothers, the delivery location and choice of child immunization differed. The choice of child immunization clinics by mothers is often based on proximity to the mother's location.

### Measurement of Postpartum Depression Symptoms

Postpartum depression was measured using the Edinburgh Postpartum Depression Scale (EPDS). It is a widely used, standardized 10-item self-administered questionnaire with scores ranging from 0 to 3 for each of the ten items (39). The positively worded items were scored in reverse order before summing up. The responses provided on the EPDS scale are then summed up resulting in a total minimum of zero (0) and a maximum of 30. The incidence of PPD was identified using a cutoff of 12 on the EPDS scale. This cutoff has also been employed in similar studies (12, 18, 40–43). Women who had a score of 13 and above were classified as experiencing probable clinical depression, while those between 0 and 12 were identified to be void of depressive symptoms.

The choice of a cutoff of 12 to indicate probable signs of depression is because it maximizes sensitivity and specificity among postpartum women (44). The mental health index (EPDS) was tested for reliability using Cronbach's Alpha. The internal consistency of similar questions within the EPDS scale as estimated by the Cronbach's Alpha in the questionnaire study was 0.82 (Table 1). This finding is similar to a Cronbach's alpha of 0.89 by Adewuya, et al. (5). This shows the scale is highly reliable and valid for measuring depression in the postpartum period.

**Table 1:** Descriptive characteristics of continuous variables.

**Variables**	**Mothers who sought care**		**Mothers who did** n**ot seek care**		**All mothers**	
	***n***	**Mean (Std Dev)**	***n***	**Mean (Std Dev)**	***n***	**Mean (Std Dev)**
EPDS	101	10.75 (5.72)	289	7.66 (5.35)	390	8.46(5.61)
Mother's age	101	30.14 (5.49)	289	30.58 (5.09)	390	30.46(5.19)
Child's age	101	4.73 (4.36)	289	4.63 (3.91)	390	4.65(4.03)
Household size	101	4.30 (1.65)	289	4.30 (1.43)	390	4.30(1.49)
Treatment cost	85	₦5,071.77 (7,589.21)	243	₦3,509.88 (13,163.53)	328	₦3,914.63 (11,979.23)
Household Income	101	₦60,702.97 (68,524.2)	289	₦53,398.27 (60,494.29)	390	₦55,290 (62,663.73)
Number of hours worked per month	101	164.75 h (53.00)	289	172.80 h (46.68)	390	170.72 h (48.46)
Household expenditure	101	₦61, 895.71 (59,269.77)	289	₦60,683.71 (53,679.49)	390	₦60,998.4 (55,109.72)

### Estimation Technique

A probit regression was employed to estimate the determinants of mental health-seeking behavior. The probit model is one of the simplest forms of the qualitative response regression model in which the dependent variable is binary, taking the form of “1” if an attribute is present and the value of “0” if that attribute is absent. In this case, the presence of the attribute is the probability of seeking healthcare while its absence is the decision not to seek mental healthcare.

### Determinant Factors/Variable Description

This study adopted the determinants of a health-seeking theory which specifies a model that identifies factors that influence the health-seeking pathway. The theory assumes that various factors determine the decision of an individual to seek (demand) healthcare and identifies broad determinants of this decision (genetics, behavioral, environmental and physical influences, medical care, and social and economic factors). In this study, the determinant factors are broadly characterized into four: individual woman, household, cost of treatment, maternal and child, and labor market characteristics.

The individual woman characteristics (*IWC*_*i*_) comprises the age (disaggregated into young mothers— <25 years; middle-aged mothers—between 25 and 40 years; and older mothers—above 40 years), religion (Christianity and Islam), and educational status of the woman which was classified into the four broad categories: no education, primary, secondary, and post-secondary education. The mental health index (*MHI*_*i*_) adopted the EPDS score to compute the presence (13–30) or absence (0–12) of depressive symptoms among the postpartum mothers. The household characteristics (*HHi*) include household size (numerical), family structure (polygamous or monogamous), and the presence or absence of a family history of PPD.

The cost of treatment (*Log*(*CTi*_*i*_)) measures the cost of mental healthcare to include the cost of consultation, medication, admission, and other cost incurred during treatment; the value was however logged as it was skewed. Based on the theory of demand, mental healthcare is also perceived as good wherein there is an inverse relationship between price (care cost) and demand for mental healthcare. The labor market characteristics (*LMC*_*i*_) comprised the employment status of the woman and her spouse (being employed or not), the number of hours worked per month, and household income (sum of wage and non-wage income).

### Method of Analysis

The data obtained were coded and analyzed using the IBM Statistical Package for Social Sciences SPSS version 21 and STATA 15, respectively. The probit regression analysis requires that one of the outcomes of the categorized variables be fixed as a reference category and the results would then be interpreted in relation to the reference category (45, 46).

### Demographic Characteristics of Respondents

Questionnaires were distributed to 520 mothers. Responses were received from 390 respondents (75% response rate) of whom 63 (16.15%) were below 25 years, 316 (81.03%) were between ages 25 and 40 years, and only 11 (2.82%) were above 40 years. On average, the mean age of the respondents was 30 years, while the mean age of the babies was about 5 months. Most of the respondents were married (91.28%). While 299 (76.67%) of the respondents had post-secondary education, only 91 (23.33%) of the women had at most secondary educational qualifications. Among the respondents, 283 (72.56%) of the women were Christians and 107 (27.44%) were Muslims. Most of the mothers were from a monogamous family structure (89.71%), though several of them still lived with extended family members, which is expected based on the cultural practice in Nigeria, especially where new mothers are expected to live with their in-laws.

Of the total women sampled, 209 (53.63%) of the mothers had a male child while 181 (46.41%) birthed male children. Based on the desirability of the gender birthed, 320 (82.05%) mothers had their desired gender of a child while 70 (17.95%) did not. The mode of delivery revealed that 63.33% of the mothers had their child through vagina delivery while 36.67% went through a cesarean section. Among the mothers, 42.3% were primiparous - first-time mothers, while 220 (56.4%) of the mothers were multiparous (had between 2 and 3 children), and only 5 (1.2%) had more than 5 children (grand-multiparous).

### Labor Market Characteristics

The labor market characteristics of the respondents revealed that 285 (73%) mothers were employed while 105 (26.7%) were unemployed. Most of the spouses (92.05%) of the mothers were employed while only 7.95% were unemployed. The mean household size was 4.3. The mothers worked for about 170 h per month. Most of the households earned less than their expenditure as the average household income was N55,290 naira while the mean household expenditure was about N61,000.

## Results

### Prevalence of Postpartum Depression and Mental Health-Seeking Behavior

The descriptive statistics of the continuous and categorical variables are presented in Tables 1, 2. The results as presented in Table 2 show that 81 out of 390 women presented symptoms of depression in the postpartum period, revealing a PPD prevalence rate of 20.8%. However, only 39.5% (32 out of 81 sufferers) sought healthcare; 60.5% of the women, who suffered from PPD, did not seek mental healthcare. Ironically, among the 309 non-sufferers of PPD, 22.3% (69 out of 309 non-suffers) also sought healthcare. Thus, among all the 101 women who sought mental healthcare, only 31.7% had depressive symptoms in the postpartum period, while 68.3% had no evidence of suffering from PPD.

**Table 2:** Descriptive characteristics of categorical variables.

**Variable**	**Categories**	**Mothers who sought care *n* (%)**	**Mothers who did not seek care *n* (%)**	**Total *n* (%)**	**df**	**Pearson chi^**2**^ (*p*-value)**
Depressive symptoms	Depressed	32 (31.68)	49 (16.96)	81 (20.77)	1	9.87 (0.00)
	Not depressed	69 (68.32)	240 (83.04)	309 (79.23)		
**Individual woman characteristics**						
Marital status	Not married	11 (10.89)	23 (7.96)	34 (8.72)	1	2.84 (0.83)
	Married	90 (89.11)	266 (92.04)	356 (91.28)		
Age group	Below 25 years	18 (17.82)	45 (15.57)	63 (16.15)	2	5.32 (0.07)
	Between 25 and 40	77 (76.24)	239 (82.70)	316 (81.03)		
	Above 40 years	6 (5.94)	5 (1.73)	11 (2.82)		
Religion	Christianity	71 (70.30)	212 (73.36)	283 (72.56)	1	0.35 (0.55)
	Islam	30 (29.70)	77 (26.64)	107 (27.44)		
Educational status	No formal education	2 (1.98)	3 (1.04)	5 (1.28)	3	4.33 (0.23)
	Primary education	2 (1.98)	5 (1.73)	7 (1.79)		
	Secondary education	27 (26.73)	52 (17.99)	79 (20.26)		
	Post-sec education	70 (69.31)	229 (76.67)	299 (76.67)		
**Household characteristics**						
Family history of PPD	Had history of PPD	31 (33.70)	55 (21.83)	86 (25.00)	1	5.06 (0.02)
	No history of PPD	61 (66.30)	197 (78.17)	256 (75.00)		
Family structure	Monogamy	90 (89.11)	260 (89.97)	350 (89.74)	1	0.06 (0.81)
	Polygamy	11 (10.89)	29 (10.03)	40 (10.26)		
Maternity leave	Yes	63 (62.38)	38 (37.62)	241 (61.79)	1	0.02 (0.89)
	No	178 (61.59)	111 (38.41)	149 (38.21)		
**Maternal and child characteristics**						
Child's gender	Male	54 (53.47)	155 (53.63)	209 (53.63)	1	0.00 (0.98)
	Female	47 (46.53)	134 (46.37)	181 (46.41)		
Mode of delivery	Vagina delivery	62 (61.39)	185 (64.01)	247 (63.33)	1	0.22 (0.64)
	Cesarean delivery	39 (38.61)	104 (35.99)	143 (36.67)		
Desired gender	Desired gender	87 (86.14)	233 (80.62)	320 (82.05)	1	1.55 (0.21)
	Not desired gender	14 (13.86)	56 (19.38)	70 (17.95)		
**Labor market characteristics**						
Husband's employment	Yes	80 (79.21)	205 (70.93)	285 (73.08)	1	2.60 (0.11)
	No	21 (20.79)	84 (29.07)	105 (26.92)		
Husband's employment	Yes	91 (90.10)	268 (92.73)	359 (92.05)	1	0.71 (0.40)
	No	10 (9.90)	21 (7.27)	31 (7.95)		
**TOTAL**	**101 (100)**	**289 (100.00)**	**390 (100)**		

It is evident from this study that both sufferers and non-sufferers of PPD sought mental healthcare. A possible reason for this is that the symptoms of PPD and other emotional symptoms experienced by mothers after delivery (such as baby blues) are quite similar. The difference is often in the duration of PPD compared to other symptoms. Symptoms of maternity blues often occur from birth to 14 days postpartum while PPD extends to a year postpartum and beyond in some cases.

In terms of the mental healthcare provider, the study revealed that most of the women (62.38%) sought healthcare through self-treatment, while 13.9 and 18.8% sought treatment from religious bodies and general hospitals, respectively. Only 5% sought healthcare from specialized neuropsychiatric hospitals. Self-treatment, in this case, included treatment from self-medication and consultation with friends and family. Reasons provided by the 60.5% of mothers who though suffered from PPD but did not seek mental healthcare included fear of dying, being stigmatized, perceived non-severity of the illness, belief that the symptoms will fade away, the perception that prayers and faith in God will sort the feeling, and fear of being tagged “a weak mother”.

The average treatment cost of PPD was about N3,914.63 as presented in Table 1. Women who sought care worked for fewer hours per month (164.8 h) compared to women who did not seek care (172.8 h). The reverse is observed for the household income. Women who sought care had an average household income of N60,702.97, compared to N53,398.27 of their counterparts who did not seek care. Furthermore, the bivariate analysis using the chi-square showed that being depressed (*p* = 0.00), women's age (*p* = 0.07), and family history of PPD (*p* = 0.05) were significantly related to the likelihood of seeking mental healthcare.

### Econometric Results

Table 3 presents the probit regression estimates of the determinants of the mental health-seeking behavior model to identify whether mothers who had experienced postpartum depression were more or less likely to seek mental healthcare. The relationship among the outcome variables (probability of seeking mental healthcare), presence of postpartum depressive symptoms (PPD), and the potential mediating variables was first estimated using the individual woman and household characteristics comprising age, marital status, religion, education, household size, and family history of PPD (Column 1), then adjusted for treatment cost and maternity leave (Column 2), maternal and child characteristics (Column 3), and then labor market characteristics (Column 4).

**Table 3:** Probit regression results of determinants of probability of seeking mental healthcare.

**Dependent variable: probability of seeking mental healthcare marginal effects (standard error)**				
**Variables**	**Model 1** ^**a**^	**Model 2** ^**b**^	**Model 3** ^**c**^	**Model 4** ^**d**^
**Mental health index**
Depression	0.44 (0.18)**	0.54 (0.25)**	0.63 (0.26)**	0.52 (0.28)*
**Individual woman characteristics**
Young mum	−0.95 (0.45)**	−1.49 (0.78)*	−1.98 (0.90)**	−1.67 (0.95)*
Middle age mum	−1.01 (0.40)**	−1.34 (0.73)*	−1.75 (0.84)**	−1.60 (0.87)*
Married	−0.49 (0.32)	−0.36 (0.52)	−0.57 (0.53)	−0.69 (0.56)
Islam	−0.04 (0.18)	0.23 (0.25)	0.28 (0.26)	0.08 (0.28)
Primary education	−0.42 (0.79)	−0.06 (0.85)	−0.44 (0.90)	−0.69 (0.94)
Secondary Education	0.20 (0.58)	0.39 (0.63)	0.14 (0.64)	0.12 (0.68)
Post-secondary Education	−0.23 (0.57)	0.06 (0.61)	−0.23 (0.62)	−0.24 (0.66)
**Household characteristics**
HHS	0.01 (0.05)	−0.00 (0.07)	0.00 (0.07)	0.03 (0.08)
Has family history of PPD	0.44 (0.17)**	0.43 (0.24)*	0.47 (0.25)*	0.42 (0.27)
**Cost of treatment characteristics**
Treatment Cost		0.06 (0.09)	0.04 (0.09)	0.04 (0.10)
Maternity Leave		0.11 (0.23)	0.16 (0.23)	0.44 (0.27)*
**Maternal and child characteristics**
Age of child			0.01 (0.03)	−0.00 (0.03)
Male child			−0.04 (0.24)	−0.11 (0.25)
Gender desired			0.77 (0.31)**	0.76 (0.32)**
Vagina delivery			0.05 (0.28)	0.09 (0.29)
**Labor market characteristics**
Employed mother				−0.42 (0.35)
Employed husband				0.32 (0.42)
log_income				0.07 (0.14)
Hours worked per month				−0.00 (0.00)
Constant	0.69 (0.82)	0.36 (1.53)	−0.46 (2.24)	−0.42 (0.35)
Observations	344	159	159	149
Pseudo R-squared	0.07	0.08	0.11	0.12
Log Likelihood	−186.08	−94.19	−90.79	−85.05
LR chi^2^ (Prob)	27.37*** (0.00)	16.68 (0.16)	23.50 (0.10)	23.87 (0.25)
Pearson chi^2^ (Prob)	126.22 (0.19)	146.82 (0.19)	163.95 (0.07)	152.24 (0.06)

“^***^”, “^**^” and “^*^” depict significance at 1, 5, and 10% levels, respectively.

*Mental Health Index, No Depression; Mother's age, Older Mother; Religion, Christianity; Education, No formal Education; Family Structure, Monogamy; Resident Extended family, None resident family member; History of PPD, No History of PPD; Maternity leave, No maternity leave; Gender of Baby, Male; Desired Gender, Gender undesirable; Mode of delivery, Cesarean section; Mother's employment status, Unemployed; Husband's Employment Status, Unemployed*.

*^a^Adjusting for the mental health index, individual woman and household characteristics*.

*^b^Adjusting for characteristics in Model 1 and cost of treatment*.

*^c^Adjusting for characteristics in Model 2 and maternal and child characteristics*.

*^d^Adjusting for characteristics in Model 3 and labor market characteristics*.

The results of the estimation as shown in Table 3 reveal that a higher incidence of PPD significantly predicts a higher probability of seeking mental healthcare among sufferers of PPD relative to non-sufferers. This effect is positive and significant across all four columns. However, the probability increases from 0.44 in the baseline estimates to 0.54 with the introduction of treatment costs and maternity leave. This likelihood further rose to 0.63 when maternal and child characteristics were incorporated into the model. The probability of seeking mental healthcare however decreased to 0.52 when labor market characteristics were integrated into the model. More severe cases of PPD might be disabling enough to propel the sufferers to seek mental healthcare.

With regards to age, the estimates show that younger (below 25 years) and middle age (between 25 and 40 years) mothers were less likely to seek mental healthcare relative to older mothers (above 40 years). Being a younger and middle-aged mother reduced the likelihood of seeking mental care by 0.95 and 1.01, respectively, relative to being an older mother. This likelihood increased to 1.49 for the younger mothers and 1.34 for middle-aged mothers when adjusted for treatment cost, 1.98 and 1.75 when adjusted for maternal and child characteristics while it reduced to 1.67 and 1.60 when labor market characteristics were introduced. The same trend, with a lower probability, is observed with middle-aged mothers in comparison with older mothers. This might be closely related to the ability of older mothers to better differentiate between PPD and the normal baby blues associated with recent childbirth, based on their experience as compared to the experience of younger mothers. Other individual woman characteristics (marital status, religion, education, and household size) were not significant determinants of MHSB.

Having a family history of PPD was significant. Mothers who had a family history of postpartum depression had a higher probability (0.44) of seeking mental healthcare. This effect remains almost the same even after controlling for the cost of treatment and maternal and child characteristics. However, having a family history of PPD became insignificant after controlling for labor market characteristics. The significance of a history of PPD in the woman's family can be because the sufferer will be encouraged to seek prompt healthcare as the invisible illness will not be misconstrued as any other illness. In addition, family members will most likely understand the symptoms and be aware of places where to seek healthcare.

The cost of treatment and maternity leave were both not significant as determinants of women seeking healthcare in the second and third columns. Across all the models, care cost was not a determinant of the decision of seeking mental healthcare. Its non-significance might reveal that regardless of the cost of treatment, women with severe cases of PPD will still seek care.

Women who had maternity leave from work had a higher likelihood of seeking mental healthcare (0.44) compared to their counterparts who did not have maternity leave. It is important to note here that maternity leave is given to women who work in formal employment, thus they can recuperate before returning to their paid employment. However, the self-employed might find it difficult to have a structured maternity leave. Having a maternity leave provides ample time for the mother to seek care before the resumption of work.

Women who desired a particular gender for their child (preferably male due to the patriarchal nature of Nigeria) were more likely to seek care (0.77) relative to those who did not have that desire. This effect was almost the same (0.76) even when labor market characteristics were included in the model. This could be due to the feeling of security of the woman, owing to the belief that if she has a male child, she has secured the love and attention of her spouse.

## Discussion

This study confirms some similarities in determinants of mental health-seeking behavior in Nigeria as compared with other countries. But there are also significant peculiarities and surprises in the behavior pattern.

The most prominent reason identified for failing to seek mental healthcare was a lack of knowledge of the symptoms of the illness, as most of them argue that *they did not recognize the symptoms as requiring health-seeking behavior*. Childbirth and child care immediately after birth are demanding both physically and mentally. Most women appear not to be able to differentiate between the challenges of childcare after birth and mental health issues. This is an outcome of low and weak mental health education. This is supported by the findings of Whitton (21) who opined that although most women suffering from PPD recognized there was something wrong, more than 80% of them did not report their symptoms to any health professional. This might be due to a lack of experience in differentiating between the demands of childcare after childbirth and mental health conditions.

In addition, mental health is associated with stigmatization. In the Nigerian case, stigmatization work in two ways. First, a new mother is expected to be strong without showing signs of weakness *while taking care of the baby*. Most women are therefore unwilling to seek care because of the stigmatization of being labeled “a weak or incapable mother” (11, 12). The second level has to do with the stigmatization of mental illness itself whether it is caused by childbirth or not.

Furthermore, many women do not seek healthcare when there is a perception of *non-severity of the symptoms*. The perceptions that “*the symptoms will go by itself* ”, and that “*there is no need to seek for healthcare*” were dominant among the mothers. In an extreme case, a respondent stated she did not want to die as her reason for not seeking care. A few mothers stated that it was a personal issue that could be handled personally “*by myself* ”. While there is a lot of support system from older family members, especially the in-laws during the period of childbirth, the mother is also careful to be seen as strong and capable of caring for her new child, especially before her in-laws. The perception of the non-severity of the symptoms felt by these mothers can be attributed to the lack of physical visible symptoms of mental illness as compared to visible symptoms of physical illnesses. This is also a reflection of the lack of awareness of mothers about PPD, its symptoms, and associated dangers. This calls for public education with a focus on mothers.

Of particular interest is the fact that while only 39.5% of depressed women sought care, 22.3% of non-sufferers also sought mental healthcare. This shows that *both sufferers and non-sufferers sought mental healthcare*. It also affirms the notion of “the worried well” by Button, et al. (24) who opined that those who seek care for mental illness were not diagnosed with the illness. The communal nature of the Nigerian society might be a reason for this where the more experienced and older members of the family are to have the capacity to diagnose an illness whether visible or not. This further indicates that regardless of the nature of the symptoms felt, there is a lot of uncertainty and difficulty in recognizing PPD and indeed other mental disorder symptoms, despite the notion by Gaynes et al. (13), Bowen, et al. (14), and Li, et al. (15) who affirm that ~10–20% of pregnant and postpartum women experience depression and /or anxiety during pregnancy or after childbirth.

In addition, it was observed that *most of the women who sought care, did so informally*; through self-medication which included being treated by themselves and/or consulting with friends and family. This was also observed by Goyal, et al. (12) who concluded that some of the mothers who had symptoms of PPD were unaware and did not perceive it as a psychological illness. The more experienced family members in Nigerian society are also more likely to proffer the expected nature, prospect, and possible solution to such illnesses. Furthermore, the nature of the healthcare system in the country is such that over 70% of healthcare expenditure is out-of-pocket. The economic condition of the country, which features a high level of poverty and income inequality, further worsened by the COVID-19 pandemic, makes it difficult to devote the financial resources required for the intensive and long-term nature of mental health treatment.

Only 5% of the women sought healthcare from a specialized neuropsychiatric hospital. This might be closely linked to the *absence of a neuropsychiatric hospital in Oyo State (the study area)*, even though a department of psychiatry exists in the University College Hospital located in the state. This finding is affirmed by Whitton (21), whose study revealed that 54% of the women who sought care spoke to friends and family and only 4% spoke to a health practitioner.

A *higher incidence of postpartum depression* among the sufferers increased the likelihood of seeking mental healthcare. This is supported by the finding of Andrade, et al. (47), Kessler, et al. (48), Tegegne and Legese (49), and Grissette, (50) who opined that the severity of illness, not the cost of treatment, motivated the demand for mental healthcare. Severe PPD necessitating seeking healthcare could be attributed to the disabling nature of severe cases of PPD, where the woman is unable to cope with the normal stresses of life, thereby leaving the patient with no choice but to seek healthcare.

Women whose family members have had a *history of PPD* were more likely to seek care. This is substantiated by Bina (51) who opined that predictors of women with PPD seeking healthcare had a history of depression. Also, since most of the women sought healthcare from family and friends, having a family history of PPD could be attributed to the family member's knowledge of the symptoms and nature of PPD and not stigmatizing the affected women about PPD being a sign of weakness. Also, family intervention and support are perceived to play strategic roles in healthcare-seeking as they increase health literacy as observed by Odaman and Ibiezugbe (32) and Smith (36). A sufferer whose family member had experienced PPD has higher chances of seeking care since such family members will be able to distinguish between symptoms of the expected maternity blues and PPD and encourage the new mother to seek mental healthcare. This is affirmed by Button, et al. (24) who opined that informal consultation with another person with whom the woman is familiar influences her decision to seek healthcare.

The non-significance of *religion* in this study contradicts the results of Gotsadze, et al. (34) who identified religion or religious beliefs as a supporting factor to seek healthcare. This finding differs from the conventional belief that religion provides support for the people. The case of mental illness is peculiar, as the cultural belief of stigmatization and the choice of the victims to seek self-treatment is likely to undermine the expected supportive roles played by religion. This finding also contradicts Olasehinde (1) who found that household members prefer to consult traditional healers, patent medicine vendors (PMVs), and religious bodies (churches/mosques) before consulting healthcare professionals (HCPs). In this study, this could be explained by the fact that *as self-selected participants already at a health facility, they had overcome any potential religion-related barriers*.

Contrary to the findings of Tegegne and Legese (49), *marital status* was not found to be a significant determinant of PPD health-seeking behavior. This suggests that the presence or absence of a spouse is inconsequential for the choice to seek care. In Northwestern China, being divorced or unmarried (non-marital status) was found to be associated with lower use of mental health services (37). Perhaps this disparity highlights the differences in cultural attitudes and contexts of marriage in Nigeria (Africa) and China. It could mean in China, the core family (husband and children) constitutes the main support to a mother. *However, in Africa, there are many informal and extended family relations, which play a significant role in supporting mothers*. This might be attributed to the fact that in the choice to seek mental healthcare in Nigeria, the extended family plays a more influential role than the nuclear family. Hence, being married or not, does not greatly impact the decision to seek care.

The non-significance of the *treatment cost* in the decision to seek mental healthcare contradicts the findings of Hjortsberg (52), Jameson (53), Muriithi (28), Olasehinde (1), and Hossain et al. (23) who observed that user costs were a major deterrent of seeking care. This finding would be intriguing if it was a community-based study, given that treatment always comes at a cost and is the commonest deterrent of demand for healthcare in general. However, this was a study of self-selected mothers who were already in a health facility; that is, *they were mothers who could afford to pay for healthcare*. Therefore, treatment cost was not an issue. Furthermore, against the perception of many, the treatment of mental illness, though long term, is often not as expensive as assumed. Unlike physical illness, mental illness requires the consistent creation of an enabling environment for the sufferers to thrive and do away with the triggers of the mental illness.

The study shows that based on *age*, young (below 25 years) and middle-aged (between 25 and 40 years) mothers were less likely to seek mental healthcare relative to older mothers (above 40 years). This is supported by the studies of Muriithi (28), Olasehinde (1) who also found that the probability of seeking professional healthcare increases with age. Thus, being a younger or middle-aged mother reduced the likelihood of seeking mental healthcare. The finding that only older mothers were likely to seek mental healthcare could be due to the previous experience of such mothers who might be likely to be able to understand the peculiarity of the symptoms they might be experiencing. This is also confirmed by Olasehinde and Olaniyan (29) who found that the adults in prime age, regardless of gender, are less likely to seek healthcare (54). This is in line with a general perception about the human life cycle: those who are in their prime years are healthier and less likely to get serious health conditions, and therefore less likely to seek healthcare.

The finding of the non-significance of *income and employment status* of both the woman and her spouse contradicts evidence from the study of Gotsadze et al. (34) in China and Bina (51) and Hansotte, (11). In contrast to the findings of Olasehinde (1) and Muriithi, (28), income did not affect a mother's decision to seek mental healthcare. This finding is expected since user cost was also observed as a non-determinant. Again, this is not surprising, given that the study participants were self-selected by already being at a health facility, meaning *they were able to afford healthcare*. Those who could not afford to pay for healthcare would not be found at a health facility. The mothers who sought healthcare possibly all came from a higher income bracket supported by employment or other forms of getting income. Thus, labor market outcomes in Nigeria are not essential factors in determining the health-seeking behaviors of sick individuals.

The *long-term nature of the treatment* (drugs and therapy) dissuaded some mothers from seeking healthcare as they stated they would rather pray about the symptoms. This could be perceived as an attitudinal barrier to not only seeking but also staying in treatment, as affirmed by Andrade et al. (47). The extended period of treating mental illness is often perceived to be accompanied by the stigmatization of continual hospital visits. This calls for the sensitization and education of mothers and the public about PPD.

It is evident from the low proportion of women who sought healthcare that there is low mental health literacy in the study area as evidenced by women with PPD who gave the reason “*It will go away on its own*”. This belief by the affected women of their experiences being part of their “destiny” is affirmed by Bruffaerts et al. (38) and Small et al. (55). It is important to promote mental health awareness among postpartum mothers. To this end, PPD should be routinely screened during postnatal and immunization clinics. In addition, there is a high level of stigmatization and discrimination associated with mothers experiencing depression in the postpartum period. This can discourage sufferers from seeking healthcare for mental illness, as affirmed by Button et al. (24), Adjorlolo et al. (56) and Goyal et al. (12). It is therefore vital to engage the community, religious, and traditional leaders to promote community sensitization efforts on mental health issues in the country.

## Limitations of the Study

The study has several limitations. First is the high non-response rate because most of the mothers were playing a dual role of filling the questionnaire and caring for the baby, as most of the respondents were from the immunization rather than postnatal clinics. This is because of the very low postnatal attendance in Nigeria. Also, most of the sampled women hardly knew the exact time of onset and duration of the depressive symptoms. Another limitation of this study that may have affected the detection rates of postpartum depression is the use of only a self-administered questionnaire to measure depression, without combining it with the clinical records of the respondents.

The study was facility-based, and as such women who did not attend either postnatal or immunization services were excluded from the study. The strengths of this study include its large sample size and the diversity of the study sample in terms of the use of private and public healthcare providers which can indicate various socioeconomic statuses.

## Conclusion

Most depressed women are unaware that they are experiencing a treatable mood disorder. The severity of mental illness, age, and family history of PPD are the main determinants of demand for mental healthcare in the study area. While having a family history of postpartum depression, the desired gender of the child and experience of severe depression increased the likelihood of seeking mental healthcare, being a younger mother reduced this possibility. Also, this study has revealed that the cost of treatment, labor market factors, and marital status did not determine the decision to seek mental healthcare.

More studies are recommended to identify determinants of the choice of mental healthcare for sufferers of postpartum depression. This study can also be replicated in the post-COVID-19 era to identify the impact of the pandemic. Furthermore, a community-based study can be conducted using combined clinical diagnosis and self-administered questionnaires.

Non-cost strategies can be implemented to encourage mothers to seek mental healthcare, as the cost of treatment is not a deterring factor. Women attending antenatal clinics should be routinely screened for possible signs of PPD to encourage prompt demand for healthcare rather than till its severity becomes disabling. Mental healthcare should be made available and integrated into maternal healthcare services provided at all levels of provision. Screening of mothers for PPD should be done during routine immunization and postnatal visits. Increasing the availability of mental healthcare facilities in the country can promote higher patronage of formal mental healthcare services.

The introduction of the National Health Insurance Scheme (NHIS) at the National level, and its adoption in individual states of the country, Nigeria, is expected to make healthcare more affordable and accessible. In addition to this scheme, access to mental health services can be achieved by increasing the health budget and specifically the proportion of the budget allocated to mental health.

## Data Availability Statement

The raw data supporting the conclusions of this article will be made available by the authors, without undue reservation.

## Ethics Statement

The studies involving human participants were reviewed and approved by University of Ibadan/University College Hospital Ethical Review Board (UI/UCH ERB) with Number NHREC/05/01/2008a in Ibadan, Oyo State, Nigeria. The patients/participants provided their written informed consent to participate in this study.

## Author Contributions

OOd prepared the research instruments, compiled the literature review, computed the theoretical framework, conducted the data collection, analysis, discussion, and interpretation of results. OOl prepared the introduction, formalized the research questions, conceptualized the study, and prepared the conclusion. SO contributed to the literature review, discussion sections, and provided technical support in the entire manuscript. All authors contributed to the preparation of the submitted version. All authors contributed to the article and approved the submitted version.

## Funding

The authors acknowledge the financial support provided by the African Economic Research Consortium (AERC), Nairobi, Kenya, the African Health Economics and Policy Association (AfHEA), and the World Health Organization Africa Region Office (WHO-AFRO).

## Conflict of Interest

SO was employed by African Centre for Global Health and Social Transformation. The remaining authors declare that the research was conducted in the absence of any commercial or financial relationships that could be construed as a potential conflict of interest.

## Publisher's Note

All claims expressed in this article are solely those of the authors and do not necessarily represent those of their affiliated organizations, or those of the publisher, the editors and the reviewers. Any product that may be evaluated in this article, or claim that may be made by its manufacturer, is not guaranteed or endorsed by the publisher.
